# Growth of semiconducting single-wall carbon nanotubes with a narrow band-gap distribution

**DOI:** 10.1038/ncomms11160

**Published:** 2016-03-30

**Authors:** Feng Zhang, Peng-Xiang Hou, Chang Liu, Bing-Wei Wang, Hua Jiang, Mao-Lin Chen, Dong-Ming Sun, Jin-Cheng Li, Hong-Tao Cong, Esko I. Kauppinen, Hui-Ming Cheng

**Affiliations:** 1 Shenyang National Laboratory for Materials Science, Advanced Carbon Division, Institute of Metal Research, Chinese Academy of Sciences, 72 Wenhua Road, Shenyang 110016, China; 2 Nano Materials Group, Department of Applied Physics and Center for New Materials, School of Science, Aalto University, PO Box 15100, FI-00076 Aalto, Finland; 3 Faculty of Science, Chemistry Department, King Abdulaziz University, Jeddah 21589, Saudi Arabia

## Abstract

The growth of high-quality semiconducting single-wall carbon nanotubes with a narrow band-gap distribution is crucial for the fabrication of high-performance electronic devices. However, the single-wall carbon nanotubes grown from traditional metal catalysts usually have diversified structures and properties. Here we design and prepare an acorn-like, partially carbon-coated cobalt nanoparticle catalyst with a uniform size and structure by the thermal reduction of a [Co(CN)_6_]^3−^ precursor adsorbed on a self-assembled block copolymer nanodomain. The inner cobalt nanoparticle functions as active catalytic phase for carbon nanotube growth, whereas the outer carbon layer prevents the aggregation of cobalt nanoparticles and ensures a perpendicular growth mode. The grown single-wall carbon nanotubes have a very narrow diameter distribution centred at 1.7 nm and a high semiconducting content of >95%. These semiconducting single-wall carbon nanotubes have a very small band-gap difference of ∼0.08 eV and show excellent thin-film transistor performance.

## Abstract (web summary)

Growth of high-quality semiconducting single-wall carbon nanotubes is crucial for high-performance devices. Here, the authors report a partially carbon-coated cobalt nanoparticle catalyst which catalyzes growth of predominantly semiconducting single-wall carbon nanotubes with a narrow band-gap distribution.

## Graphical abstract

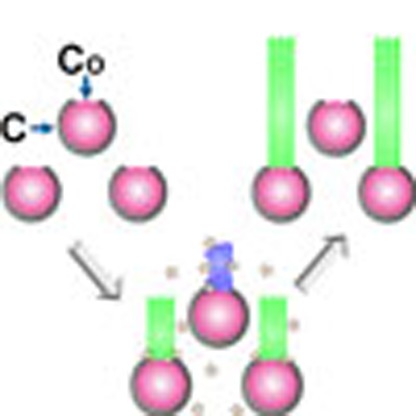

Single-wall carbon nanotubes (SWCNTs) have a small diameter, high carrier mobility, tunable band-gap and good stability^1^,^2^,^3^. Therefore, they are considered to be an ideal channel material for high-performance field effect transistors (FETs), integrated circuits and related electronic devices^4^,^5^,^6^. However, slight changes in the diameter and chiral angle of a SWCNT lead to the change of its type of electrical conductivity, that is, semiconducting to metallic or *vice versa*. As a result, as-prepared SWCNTs are usually a mixture of semiconducting and metallic nanotubes. In recent times, great efforts have been devoted to the selective preparation of pure semiconducting SWCNTs (s-SWCNTs) in large scale for their potential use in new-generation integrated circuits and notable progress has been made. One way is to separate SWCNTs by postsynthesis chemical and physical treatments^7^,^8^,^9^,^10^. Although high-purity s-SWCNTs can be obtained, this approach suffers from drawbacks that contamination and defects are inevitably introduced and the resultant SWCNTs are usually shortened. The other way is to synthesize s-SWCNTs directly by using metal nanoalloy as catalyst^11^,^12^ or *in situ* selective etching during growth based on the principle that metallic SWCNTs (m-SWCNTs) are chemically more active than s-SWCNTs^13^,^14^. For example, Zhang and colleagues^15^ grew s-SWCNT arrays by applying ultraviolet irradiation and subsequent photochemical reactions; Liu and colleagues^16^ prepared SWCNTs by using a mixture of ethanol and methanol as both carbon source and oxidant precursor; Cheng and colleagues^17^,^18^ synthesized s-SWCNTs by introducing oxygen or hydrogen as etchants; and Li and colleagues^19^ grew s-SWCNTs from a metal catalyst loaded on CeO_2_ supports that can release oxygen for selective etching. In these above studies, high-quality s-SWCNTs with purities higher than 90% were obtained. However, little attention has been paid to the band-gap uniformity of the s-SWCNTs obtained. In fact, the diameters of these s-SWCNTs are usually distributed over a wide range. According to theoretical and experimental studies, the band-gap of s-SWCNT is inversely proportional to their diameter^2^. It is only by using s-SWCNTs with a narrow band-gap range that devices with a uniform and stable performance can be fabricated. From this point of view, it is most desirable to grow SWCNTs with uniform chirality. Progress has been made on the controlled growth of SWCNTs by catalyst engineering^20^,^21^,^22^. Harutyunyan *et al.*^20^ first achieved preferential growth of m-SWCNTs by modifying the morphology and coarsening behaviour of catalyst nanoparticles. Scott *et al.*^23^ synthesized short (5, 5) CNTs from a corannulene seed by stepwise chemical method and by Diels–Alder reaction^24^. Theoretically, Ding *et al.*^25^ proposed that the growth rate of an SWCNT is proportional to its chiral angle, whereas Artyukhov *et al.*^26^ pointed out that the naturally enriched near-armchair SWCNTs can be attributed to a ‘compromise' of the kinetic and thermodynamic growth trends for a rigid state catalyst. Very recently, high-purity single-chirality SWCNTs were grown from high-melting-point, W-containing alloy nanoparticles^21^,^22^, as well as growth seeds derived from molecular precursors^27^. However, the enriched (12, 6)^21^, (6, 6)^27^ and so on SWCNTs are metallic, whereas the content of (16, 0) s-SWCNTs is ∼80% (ref. 22).

To obtain s-SWCNTs with a narrow diameter distribution and a small range of band-gap, it is essential to use uniform catalyst nanoparticles. However, it was found that even with a similar initial catalyst nanoparticle size, the grown SWCNTs may have different diameters^28^,^29^. There are mainly two reasons for this phenomenon: the first is that catalyst nanoparticles tend to aggregate, to form larger ones during the growth of SWCNTs at high temperatures; second, SWCNTs may grow following either the ‘tangential' mode (with diameter equal to the nanoparticle size)^28^ or the ‘perpendicular' mode (with diameter smaller than the nanoparticle size)^28^. Therefore, to achieve well-controlled growth of SWCNTs, it is crucial to prevent the aggregation of catalyst nanoparticles and to control their mode of nucleation and growth. In this study, we design and prepare an acorn-like bicomponent catalyst, that is, Co nanoparticle with a partial carbon coating layer. The inner Co nanoparticle functions as active catalytic phase for SWCNT growth, whereas the outer carbon layer prevents the aggregation of Co nanoparticles and ensures the perpendicular growth of SWCNT from the catalyst. This acorn-like catalyst with uniform size and structure is prepared using a block copolymer (BCP) self-assembly technique^30^. The SWCNTs grow from the catalyst have a very narrow diameter distribution. Based on the principle that m-SWCNTs are chemically more reactive than s-SWCNTs with similar diameters due to the smaller ionization potential of the former^31^,^32^, hydrogen is introduced as an etchant and m-SWCNTs are selectively removed *in situ*. As a result, high-purity (>95%) s-SWCNTs with a narrow range of band gaps (<0.08 eV) are obtained, which show an excellent thin-film transistor (TFT) performance.

## Results

### Preparation of Co nanoparticle partially coated with carbon

**Figure 1:**
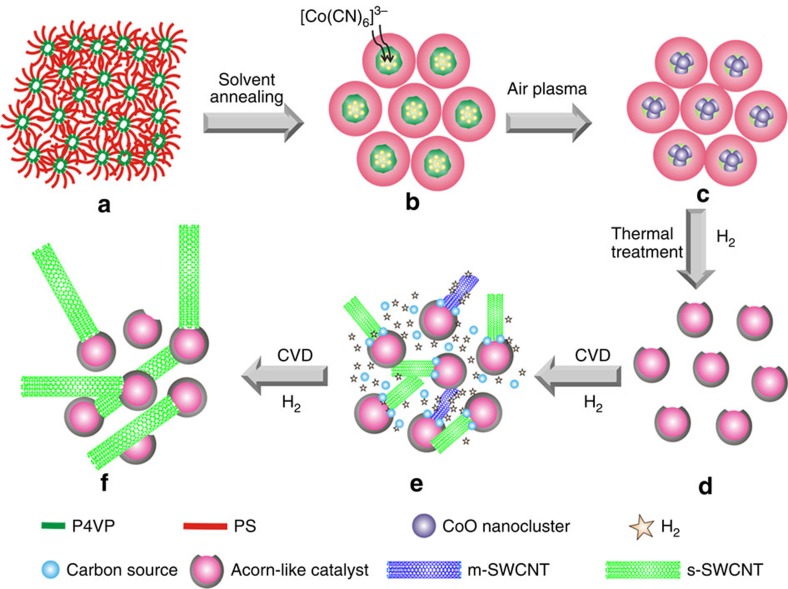
Schematic showing the formation of carbon-coated Co nanoparticles and the growth of s-SWCNTs. (**a**) A self-assembled PS-b-P4VP film. (**b**) Formation of phase-separated nanodomains and adsorption of [Co(CN)_6_]^3−^ catalyst precursors. (**c**) CoO nanoclusters partially surrounded by residual polymer. (**d**) Co nanoparticles partially coated with carbon. (**e**) Nucleation and growth of SWCNTs with a narrow diameter distribution grown from the partially carbon-coated Co nanoparticles and *in situ* etching of m-SWCNTs. (**f**) s-SWCNTs with a narrow band-gap range.

A schematic showing the preparation of the Co nanoparticles partially coated with carbon is shown in Fig. 1. Details of catalyst preparation are described in Methods. In brief, an asymmetric poly-(styrene-block-4-vinylpyridine) (PS-b-P4VP) film (Fig. 1a) was self-assembled into vertical P4VP nanocylinders by placing it in a toluene/tetrahydrofuran (THF) mixture vapor annealing for 24 h. The formed nanodomains, consisting of hydrophilic vertical P4VP nanocylinders enclosed by a hydrophobic PS matrix, were then immersed in an acidic aqueous solution of K_3_[Co(CN)_6_] (Fig. 1b). The protonated pyridinic nitrogen sites in the P4VP nanocylinders attracted anionic Co complexes as [Co(CN)_6_]^3−^, after which the material was treated in an air plasma to oxidize the polymer. As the Co precursor was anchored at one end of the nanodomains, CoO nanoclusters partially encapsulated by a thin polymer layer were obtained (Fig. 1c). It is worth noting that we intentionally used a relatively weak plasma treatment to remove the polymer in a controlled manner. The PS-b-P4VP used, PS40000-b-P4VP5600, has a high PS to P4VP mass ratio (∼7), which also makes it difficult to completely remove the polymer. Furthermore, owing to the similar size of the self-assembled nanodomains, the resulting CoO clusters have a uniform size and exposed surface area. In subsequent heating in a H_2_ atmosphere, the CoO nanoclusters were reduced to Co and aggregated into Co nanoparticles, and the residual polymer was carbonized to produce Co catalysts that were partially coated with carbon; thus, the acorn-like bicomponent catalyst is obtained (Fig. 1d). SWCNTs with a uniform diameter (Fig. 1e) were grown from these catalyst particles by the chemical vapour deposition (CVD) of ethanol. When H_2_ was introduced as an *in situ* growth etchant, m-SWCNTs with higher chemical reactivity were selectively removed and s-SWCNTs with a narrow range of band-gap were obtained (Fig. 1f).

### Characterization of the partially coated Co nanoparticles

**Figure 2:**
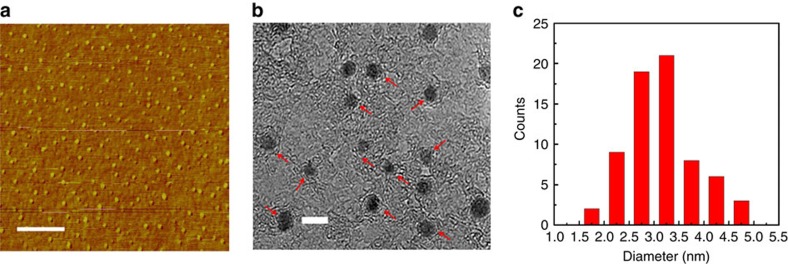
Morphology and diameter distribution of the Co nanoparticle partially coated with carbon. Typical (**a**) AFM and (**b**) TEM images of the Co nanoparticles partially coated with carbon, showing that the nanoparticles are monodispersed and uniform in size. The red arrows indicate Co nanoparticles partially coated by a carbon layer. Scale bar, 400 nm (**a**) and 5 nm (**b**). (**c**) A histogram showing the diameter distribution of the nanoparticles based on TEM observations.

To understand the structure of the catalyst synthesized using this method, atomic force microscopy (AFM) and transmission electron microscopy (TEM) characterization were performed. An AFM image (Fig. 2a) shows that the catalyst particles are well-dispersed and uniformly distributed on the substrate, whereas the TEM image (Fig. 2b) verifies the uniform size of the monodispersed Co nanoparticles. These nanoparticles were observed to be partially coated with a carbon layer (indicated by red arrows in Fig. 2b). High-resolution TEM images (Supplementary Fig. 1a–c) clearly demonstrate the partially carbon-coated Co nanoparticle structure of the catalyst. More detailed characterizations of the catalysts are reported in Methods. The sizes of ∼130 nanoparticles were measured from TEM images and the resultant histogram of their diameters is shown in Fig. 2c. It can be seen that most of the nanoparticles (>90%) have diameters in the range of 2.5–4.5 nm, with a mean diameter of 3.1 nm. These uniform and monodispersed Co catalyst particles partially coated with carbon are critical for the growth of SWCNTs with a narrow diameter distribution.

### Structure characterization of SWCNTs

**Figure 3:**
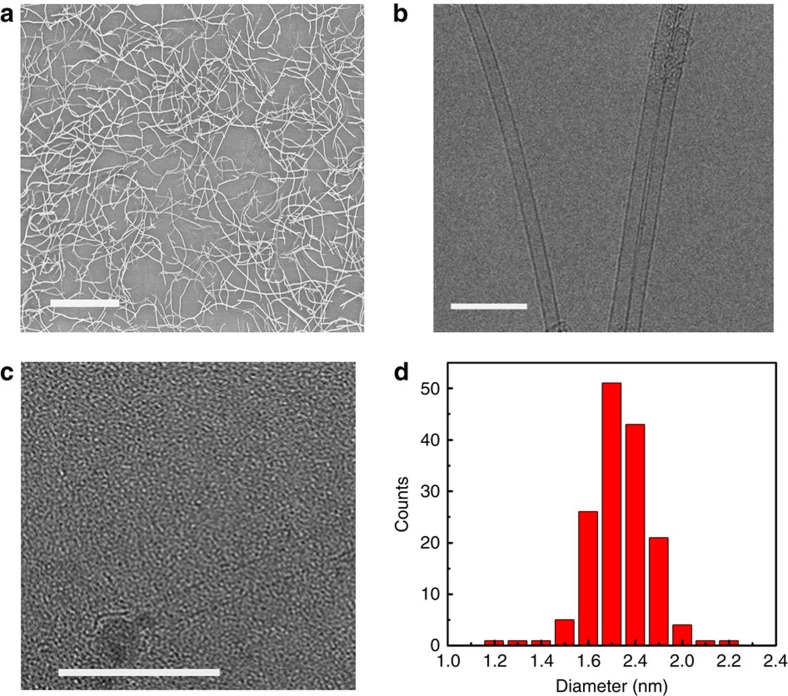
SEM and TEM observations of the as-prepared SWCNTs. SEM (**a**) and TEM (**b**,**c**) image of the as-prepared SWCNTs. Scale bar, 10 μm (**a**) and 10 nm (**b**,**c**). (**d**) Diameter distribution of the SWCNTs based on TEM observations.

SWCNTs were synthesized by CVD at 700 °C using the carbon-coated Co particles as catalyst, alcohol as carbon source and hydrogen as carrier gas. Details of the SWCNT synthesis procedure and characterization are described in Methods. Scanning electron microscope (SEM) image of Fig. 3a shows that carbon nanotubes with a length of 10 μm are randomly dispersed on the substrate. TEM observations show that the SWCNTs obtained are isolated and straight (Fig. 3b), suggesting that the catalyst has a good catalytic activity for SWCNT growth even at the relatively low temperature of 700 °C. A SWCNT grown from a partially carbon-coated catalyst nanoparticle was clearly observed (Fig. 3c). The diameters of 130 SWCNTs were measured from TEM images and the resultant histogram is shown in Fig. 3d. The diameters are narrowly distributed in the range of 1.6–1.9 nm with a mean diameter of 1.7 nm. This diameter distribution is much narrower than most previously reported results^17^,^33^. In addition, the SWCNTs are isolated rather than in bundles, which can be attributed to the monodispersed Co catalysts used.

### Characterization by Raman and absorption spectroscopy

**Figure 4:**
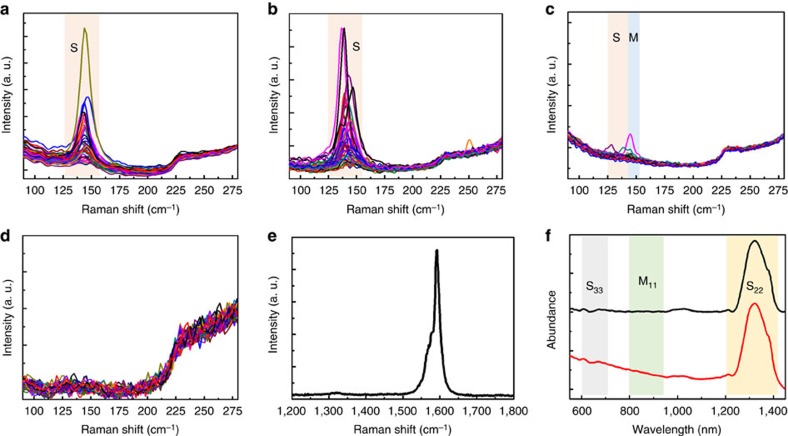
Electrical type of the SWCNTs. Electrical type characterized by multi-wavelength Raman spectroscopy and ultraviolet/visible/near-infrared absorption spectroscopy. Raman spectra of the SWCNTs measured with (**a**) 532, (**b**) 633, (**c**) 785 and (**d**) 488 nm lasers. The regions corresponding to semiconducting and metallic transitions are labelled as S (pink) and M (blue), respectively. (**e**) D and G band excited with a 633-nm laser. (**f**) Ultraviolet/visible/near-infrared absorption spectrum of an aqueous dispersion collected from 125 SWCNT samples: as-collected curve (red line) and background-subtracted curve (black line).

We further characterized the SWCNTs using Raman spectroscopy with excitation laser wavelengths of 488, 532, 633 and 785 nm. Figure 4a–d show the Raman spectra, where the radial breathing mode (RBM) peaks originating from m- and s-SWCNTs are highlighted according to the Kataura plot^34^. In Fig. 4a, sharp peaks narrowly centred at ∼141 cm^−1^ are shown with an excitation wavelength of 532 nm (2.33 eV) and these peaks originate from s-SWCNTs. Figure 4b shows the Raman spectra excited with a laser wavelength of 633 nm (1.96 eV) and the RBM peaks originating from s-SWCNTs are located in a narrow range around ∼141 cm^−1^. Raman spectra excited by a 785 nm (1.58 eV) laser (Fig. 4c) shows very few weak RBM peaks at ∼141 cm^−1^ originating from metallic tubes. No RBM Raman peak is detected with the 488-nm laser (2.54 eV) (Fig. 4d). All the excited Raman RBM signals under these four laser wavelengths are centred at ∼141 cm^−1^ and most can be assigned to s-SWCNTs, suggesting that the sample contains s-SWCNTs with a narrow diameter distribution. Using the inverse relationship between RBM peak frequency (*ω*_RBM_) and tube diameter (*d*_t_) (*d*_t_=235.9/(*ω*_RBM_−5.5))^35^, the calculated diameters of the SWCNTs are around 1.7 nm, which is in good agreement with the TEM observations. The content of s-SWCNTs in our sample is calculated to be ∼98% based on the number of the metallic and semiconducting RBM peaks averaged from 160 Raman spectra^19^,^36^. The G-band of the Raman spectra (Fig. 4e) show an obvious Lorentzian line shape, which is characteristic of s-SWCNTs^37^ and this further verifies the effective enrichment of semiconducting nanotubes. As the diameters of the SWCNTs are mostly in the range of 1.6–1.9 nm, these s-SWCNTs have an extremely small band-gap difference of 0.08 eV (ref. 38). The ultraviolet/visible/near-infrared absorption spectrum of an aqueous SWCNT dispersion collected from 125 SWCNT samples (for more details, see Methods and Supplementary Fig. 2) is shown in Fig. 4f. One intense narrow peak and two weak peaks distributed in the range of 1,200–1,430 nm and 600–715 nm, respectively, were detected, corresponding to the second and third van Hove singularity transition of s-SWCNTs (*S*_22_ and *S*_33_) with diameter distributions of 1.6–1.9 nm (refs 34, 38). No obvious first van Hove singularity transition of m-SWCNTs (800–950 nm) was detected by the ultraviolet/visible/near-infrared, confirming the high purity of s-SWCNTs in our sample. Quantitatively, the content of s-SWCNTs in the sample was calculated to be ∼ 99% (ref. 39).

### Conductivity characterization by electron diffraction

**Figure 5:**
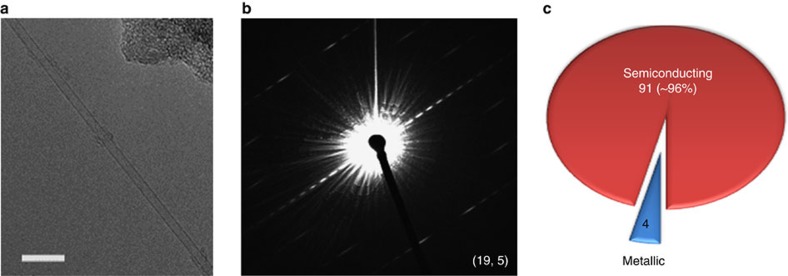
Electron diffraction of the SWCNTs. (**a**) Typical TEM image of an isolated SWCNT. Scale bar, 10 nm. (**b**) Electron diffraction pattern of the SWCNT shown in **a**, the chirality of which is assigned as (19, 5). (**c**) A pie chart showing the content of s- and m-SWCNTs based on electron diffraction measurements of a total of 95 SWCNTs.

Electron diffraction analysis is an effective method for directly determining the chiral indices (*n*, *m*) of SWCNTs^40^. To further confirm the enrichment of s-SWCNTs in our sample, we transferred the SWCNTs from a Si substrate to a TEM grid (for details see Methods) and carried out electron diffraction using a TEM (JEOL JEM-2200FS 2 × CS corrected) operated at 80 kV with a Gatan 794 multiscan charge-coupled device camera (1 k × 1 k). Figure 5a shows a freestanding SWCNT and its corresponding electron diffraction pattern is shown in Fig. 5b. In addition to the bright spot at the centre caused by the direct electron beam, the diffraction pattern mainly consists of a set of parallel diffracted layer lines, which are separated by certain distances from the equatorial layer line at the centre. By analysing the layer line distance, the chiral index of this SWCNT was determined to be (19, 5) with a tube diameter of 1.72 nm. The chiral indexes of total 95 SWCNTs were assigned by using this electron diffraction method (Supplementary Figs 3 and 4). The resultant numbers of s- and m-SWCNTs are shown in Fig. 5c. The s-SWCNT content reaches ∼96%, which verifies the enrichment of s-SWCNTs in our sample.

### Electrical performance of SWCNT TFTs

**Figure 6:**
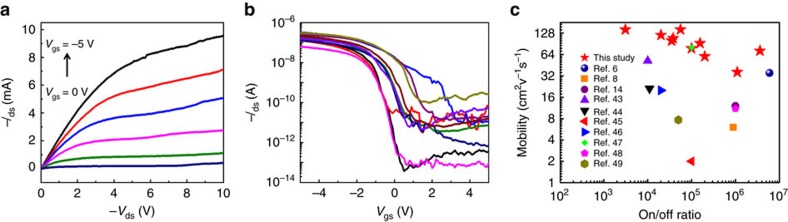
Electrical performance of the SWCNT TFTs. (**a**) Output (*I*_ds_−*V*_ds_) characteristics of the same device measured at various *V*_gs_ from −5 to 0 V in 1 V step. *L*_ch_=200 μm, *W*_ch_=100 μm. (**b**) Transfer characteristics of 10 SWCNT TFTs. (**c**) A comparison of the mobility performance of our SWCNT TFTs with those previously reported.

Electrical measurements can provide direct evidence of the transport properties of SWCNTs^41^,^42^. Thus, TFT devices based on the SWCNTs with a growth time of 25 min were fabricated (for more details, see Methods). Supplementary Fig. 5 shows a typical SEM image of a long channel bottom-gated SWCNT TFT. It can be seen that the SWCNTs are pure and randomly distributed. The channel width and length of the TFTs are 100 and 200 μm, respectively. Figure 6a shows typical output characteristics (*I*_ds_−*V*_ds_)of the TFTs, which indicates the formation of ohmic contacts between SWCNTs and metal eletrode. Figure 6b shows transfer characteristics (*I*_ds_−*V*_gs_) of ten devices with the same channel geometry (*L*_ch_=200 μm, *W*_ch_=100 μm); simultaneously, high on/off ratios of 3.1 × 10^3^–3.6 × 10^6^ and high carrier mobilities of 36–143 cm^2^ V^−1^ s^−1^, as evaluated by the standard formula^6^, were demonstrated. Remarkably, the mean carrier mobility is as high as 95.2 cm^2^ V^−1^ s^−1^. Furthermore, as shown in Fig. 6c, when compared with the previously reported SWCNT-based TFTs^6^,^8^,^18^,^43^,^44^,^45^,^46^,^47^,^48^,^49^, including the TFTs fabricated using chirality-enriched nanotubes^46^, our TFTs show obviously superior performance, in terms of both high current on/off ratio and high carrier mobility, which further verifies the high quality and high s-SWCNTs content of our sample. We further constructed a series of bottom-gate TFTs from the grown s-SWCNTs with *L*_ch_ ranging from 20 to 200 μm (Supplementary Fig. 6). It was found that on/off ratios higher than 10^5^ were obtained for the TFTs with *L*_ch_ longer than 40 μm, and even for the devices with a *L*_ch_ value of 20 μm, the on/off ratios were still around 10^2^. TFTs with a short channel length of 1.5 μm were also fabricated by electron-beam lithography (EBL) using the same sample. High on/off ratios and high carrier mobilities were demonstrated for the majority of the devices (Supplementary Fig. 7), although a few TFTs showing current on/off ratios <5 caused by the short circuiting of m-SWCNTs were also detected.

## Discussion

We attribute this selective growth of s-SWCNTs to both novel catalyst and *in situ* growth selective etching. As mentioned above, we prepared an acorn-like, partially carbon-coated Co nanoparticle catalyst with a narrow diameter distribution by a BCP self-assembly method. The [Co(CN)_6_]^3−^ catalyst precursor was absorbed on the phase-separated P4VP nanodomains so that only part of the Co nanoparticle is exposed and serves as the catalyst for SWCNT growth following a perpendicular mode (see Fig. 1). As shown in Fig. 3c, SWCNTs were observed to grow from the partially carbon-coated Co nanoparticles via a perpendicular growth mode. As the Co nanoparticles prepared by the copolymer self-assembly method have a narrow range of diameters, the exposed Co active catalyst obtained after polymer wrapping and partial etching processes should have an even smaller size difference. As a consequence, the SWCNTs grown on the exposed Co regions have an extremely narrow diameter distribution. The hydrogen used as carrier gas during the CVD growth also serves as an etchant, that is, m-SWCNTs can be preferentially removed by H radicals decomposed from H_2_ at 700 °C in the presence of Co^50^. The narrow diameter distribution of the SWCNTs guarantees efficient selective etching of m-SWCNTs, as the chemical stability of SWCNTs is determined by both their electrical type and diameter^14^,^16^,^17^,^31^,^32^,^51^,^52^. Therefore, high purity (>95%) s-SWCNTs with a uniform diameter and band-gap are obtained.

To verify the above controlled growth mechanism, we further performed a series of comparative experiments. First, fully exposed Co catalyst particles were prepared by omitting the solvent annealing process and a thermal treatment at 750 °C in air for 5 min was added to completely remove the polymer (see Methods). These Co catalysts were used for growing SWCNTs. The Raman spectra (Supplementary Fig. 8) of the obtained sample show a broad RBM peak distribution, originating from both s-and m-SWCNTs according to the Kataura plot^34^. This result proves that the partial carbon coating of the Co nanoparticles plays a key role in growing SWCNTs with uniform diameters. Following this controlled growth mechanism, it may be feasible to tune the diameter of SWCNTs by controlling the exposed area of the Co nanoparticles. We therefore tried to enlarge the exposed area by performing heat treatment in a H_2_ atmosphere at 800 °C for 10 min and then used the resulting material for SWCNT growth (Methods). Supplementary Fig. 9 shows the Raman spectra of the SWCNTs obtained. It can be seen that the RBM peaks are distributed in the range of 110–120 cm^−1^, corresponding to nanotube diameters of 2.0–2.2 nm. Based on the Raman spectra from three laser wavelengths, the content of s-SWCNT was calculated to be ∼96% (refs 19, 34, 36). This result further verifies the growth mechanism of s-SWCNTs from the Co catalyst partially coated with carbon.

In summary, acorn-like partially carbon-coated Co nanoparticles with a mean diameter of 3.1 nm were prepared by a BCP self-assembly and subsequent partial oxidation approach. The bicomponent nanoparticles were used as a catalyst for growing SWCNTs by CVD. SWCNTs with a narrow and tunable diameter distribution were synthesized in a hydrogen atmosphere following a perpendicular growth mode. It was found that a high content (>95%) of s-SWCNTs with a narrow diameter distribution was obtained. The mean diameter of the s-SWCNTs could be tuned to be ∼1.7 or 2.1 nm by controlling the carbon coverage of the Co catalyst. The band-gap range of the s-SWCNTs was <0.08 eV, due to their very narrow diameter distribution. The TFTs fabricated using these s-SWCNTs showed an excellent electrical performance. This combined catalyst design and *in situ* etching approach may pave the way for chirality-controlled growth of SWCNTs.

## Methods

### Preparation of the partially carbon-coated Co nanoparticles

A BCP self-assembly technique was used to prepare Co nanoparticles with a uniform diameter^53^,^54^. Briefly, 0.25 wt% PS-b-P4VPs (Mn: 40,000 g mol^−1^ for PS and 5,600 g mol^−1^ for P4VP, Polymer Source Inc.) were dissolved in a toluene/THF=3/1 (wt ratio) solution and electromagnetically stirred for 2 h in a 70 °C water bath. The solution was then spin-coated (Spin Master100) onto a Si/SiO_2_ (300 nm thick) substrate at 5,000 r.p.m. for 2 min. The substrate had previously been treated in a sulfuric acid/hydrogen peroxide (5/1 in wt ratio) solution and ultrasonicated in ethanol and acetone for 15 min. The BCP thin films were then annealed under a saturated vapour of toluene/THF (1/4 vol ratio) solution, after which they were immersed in a 1 mmol ^l−^ K_3_[Co(CN)_6_] (98%, J&K) acidic aqueous solution for 3 min, to allow the adsorption of [Co(CN)_6_]^3−^, followed by washing three times in de-ionized water. The BCP films were then dried at 60 °C for 20 min followed by exposure to air plasma for 10 min, to remove the polymer covered on the top of particles. The CoO nanoclusters were reduced under a hydrogen atmosphere with a flow rate of 200 s.c.c.m. at 500 °C for 5 min, subsequently heated up to 700 °C for 5 min, to obtain Co nanoparticles that were partially coated with carbon.

### Synthesis of SWCNTs

For the growth of SWCNTs, a Si/SiO_2_ substrate with catalyst dispersed on the surface was placed at the low-temperature zone (end) of a quartz tube reactor (25 mm in diameter) inserted into a horizontal tube furnace. When the furnace temperature reached 700 °C, the Si substrate was put into the centre of the reactor. At the same time, a 75 s.c.c.m. argon flow through an ethanol bubbler (in a 35 °C water bath) and a 200 s.c.c.m. hydrogen flow were introduced into the reactor for 10–25 min, to grow SWCNTs. The furnace was then cooled to room temperature under the protection of the Ar/H_2_ flow.

### Characterizations of the catalysts and SWCNTs

An AFM (Digital Instruments Multiple Mode SPM Nanoscope IIIa, operated at tapping-mode), an SEM (FEI XL30 S-FEG, operated at 1 kV), a TEM, (FEI Tecnai F20, 200 kV; JEOL JEM-2200FS 2 × CS corrected TEM, 80 kV) and a micro-Raman spectroscope (Jobin Yvon HR800, excited by 488, 532, 633 and 785 nm He-Ne laser with laser spot size of ∼1 μm^2^, in line mapping mode) were used to characterize the SWCNTs. For Raman characterization, more than 40 locations in each sample were measured randomly by moving the laser spot in 10 μm steps. For TEM observation, we used the transfer procedure developed by Jiao *et al.*^55^,^56^ with a slight modification. Briefly, a polymethylmethacrylate (PMMA) solution (AR-P 679, molecular weight=950 K, 4 wt.% in ethyl lactate, Allresist) was first spin-coated (3,000 r.p.m. for 1 min) onto the substrate on which the SWCNTs were grown. The substrate was then baked at 80 °C for 4 h in an oven. The PMMA solidified and formed a thin film with SWCNTs embedded in it. It was then put into a freshly prepared NaOH aqueous solution (5 M) with a temperature of 80 °C and kept there for 100 min. The slight etching of the SiO_2_ surface by the hot NaOH solution released the PMMA film from the substrate^57^. Finally, the PMMA film embedded with SWCNTs was peeled off and attached to a Cu grid for TEM characterization after the PMMA was removed in an acetone bath^58^. For ultraviolet/visible/near-infrared absorption characterization, total 125 Si substrates (10 mm × 10 mm) with SWCNTs grown were inmmersed into a 5-ml 1 wt% sodium benzenesulfonate (SDS) D_2_O solution (one by one), followed by ultrasonic treatment for 5 min, to detach the SWCNTs from the substrate and to disperse in the D_2_O solution.

### Calculation of the s-SWCNT content from absorption spectrum

According to the tight-binding model^34^, the band gaps of s-SWCNTs and m-SWCNTs are dependent on their diameters as follows^38^:


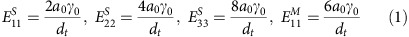


*a*_0_ is the C–C band distance and *γ*_0_ is interaction energy between neighbouring C atoms.

In this study, the diameter of the SWCNTs is distributed in the range of 1.6–1.9 nm. Thus, their absorption spectra peaks of *S*_11_, *S*_22_, *S*_33_ and *M*_11_ are calculated to be at 2,408–2,860 nm, 1,204–1,430 nm, 602–715 nm and 803–953 nm, respectively. The absorption peaks of *S*_11_ cannot be detected due to the strong noise absorption from the solvent at corresponding wavelength. Therefore, the content of s-SWCNTs was calculated by using absorption peak areas of *M*_11_ and *S*_22_ with the following formula:


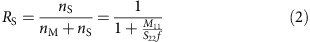


Where *R*_S_ is the content of s-SWCNTs in the sample, *n*_M_ and *n*_S_ are the numbers of m- and s-SWCNTs, *M*_11_ and *S*_22_ are the areas of the *M*_11_ or *S*_22_ peaks measured, and *f* is the absorption coefficient, here 1.24 according to the SWCNT diameter distribution. After baseline subtraction, the peak areas of *S*_22_ and *M*_11_ were measured to be 3.81 and 0.0265, respectively. Thus, the content of s-SWCNTs in our samples was calculated to be ∼99% (ref. 39).

### Fabrication of SWCNT TFTs

Bottom-gate TFTs based on our as-grown SWCNT network were fabricated on Si substrates with a thermally grown SiO_2_ layer (300 nm) as a gate dielectric. The bottom-gate electrode (Ti/Au: 10/50 nm) was deposited by electron-beam evaporation after the SiO_2_ layer on the backside of the wafer was etched by reactive ion etching. Source and drain electrodes (Ti/Au: 10/50 nm) were fabricated by standard photolithography, electron-beam evaporation and lift-off processes. Subsequently, SWCNTs outside the channel area were removed by oxygen plasma. For the fabrication of TFTs with short channel lengths, the patterning of electrodes and channel was realized by EBL. For details, the source and drain electrodes (Ti/Au: 5/50 nm) on Si/SiO_2_ substrates with grown SWCNT network were fabricated by EBL, electron-beam evaporation and lift-off processing. Next, the SWCNTs outside the channel area were removed by EBL and oxygen plasma etching. Electrical measurements were conducted using a semiconductor analyser (Agilent B1500A) at ambient conditions.

### Preparation of exposed Co catalyst and growth of SWCNTs

For comparison, a parallel experiment was conducted by using completely exposed Co nanoparticles as the catalyst to grow SWCNTs. The catalyst was prepared by omitting the solvent annealing step to obtain a uniform BCP film without microphase separation. We treated the material in air at 750 °C for 5 min to remove the carbon coating, to obtain uncoated Co nanoparticles. The fully exposed Co catalyst was used to grow SWCNTs with the same CVD conditions described in Methods.

### Synthesis of SWCNTs with a tunable mean diameter

To tune the mean diameter of SWCNTs, we prepared carbon-coated Co catalyst with a larger area of exposed Co by changing the thermal reduction process from 500 °C for 5 min to 700 °C for 10 min and to 800 °C for 10 min under H_2_ with a flow rate of 200 s.c.c.m. The catalyst was used to grow SWCNTs with identical CVD growth conditions as described in Methods. The result of Raman characterization is shown in Supplementary Fig. 9. These SWCNTs have a mean diameter of 2.1 nm and the content of semiconducting nanotubes was calculated to be higher than 96%.

## Additional information

**How to cite this article:** Zhang, F. *et al.* Growth of semiconducting single-wall carbon nanotubes with a narrow band-gap distribution. *Nat. Commun.* 7:11160 doi: 10.1038/ncomms11160 (2016).

## Supplementary Material

Supplementary Information Supplementary Figures 1-9
