# Corrigendum: Growth of semiconducting single-wall carbon nanotubes with a narrow band-gap distribution

**DOI:** 10.1038/ncomms11563

**Published:** 2016-04-27

**Authors:** Feng Zhang, Peng-Xiang Hou, Chang Liu, Bing-Wei Wang, Hua Jiang, Mao-Lin Chen, Dong-Ming Sun, Jin-Cheng Li, Hong-Tao Cong, Esko I. Kauppinen, Hui-Ming Cheng

Nature Communications 7: Article number: 11160 10.1038/ncomms11160 (2016); Published 03302016; Updated 04272016

This Article contains an error in Fig. 6. In Fig. 6a, the units on the y-axis should be ‘μA' not ‘mA'.

