# Supplementary material for: Growth of semiconducting single-wall carbon nanotubes with a narrow band-gap distribution
Source: Nat Commun. 2016 Mar 30;7:11160. doi: 10.1038/ncomms11160 (PMC4820937; doi:10.1038/ncomms11160)
Supplement: Supplementary Information — Supplementary Figures 1-9 [file ncomms11160-s1.pdf]

Supplementary Figures

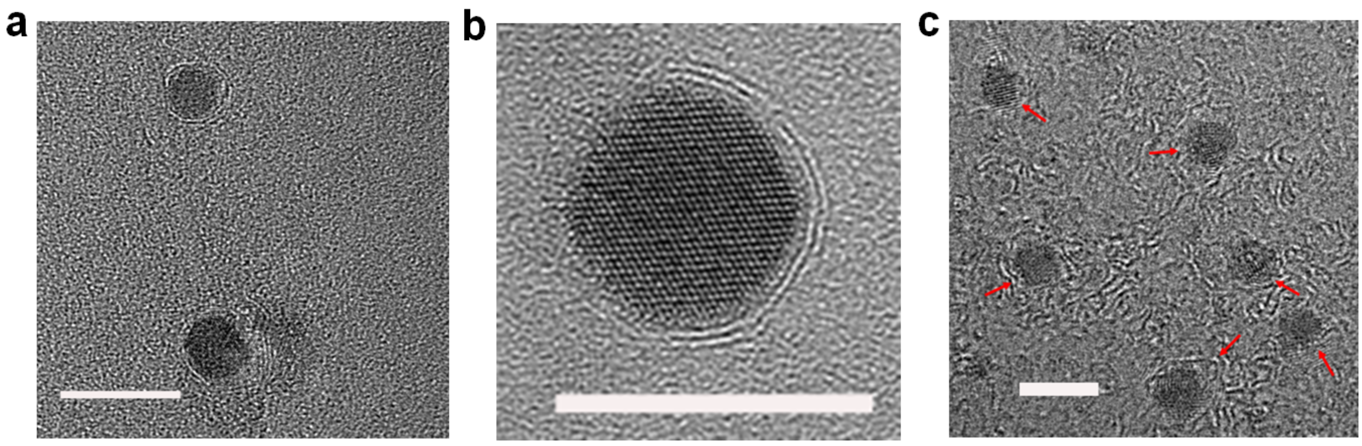

Supplementary Figure 1. Catalyst with an acorn-like structure. Transmission electron microscopy (TEM) observation of catalysts with an acorn-like catalyst structure showing that Co nanoparticles were partially coated by a carbon layer. (**a** and **b**) The catalysts were directly deposited on a SiN grid for TEM observation. Scale bars, 10 nm. (**c**) The catalysts were transferred from Si substrates to a Cu grid for TEM observation. Scale bar, 5 nm. In order to clearly observe the acorn-like Co catalyst, we deposited the catalyst nanoparticles on a SiN grid and performed identical pretreatment process as described in the main text. Then, the sample was subjected to TEM observations directly. We note that the catalyst nanoparticles thus obtained on the SiN grid are larger than those on a Si substrate. However, the structure of the acorn-like Co catalysts is the same.

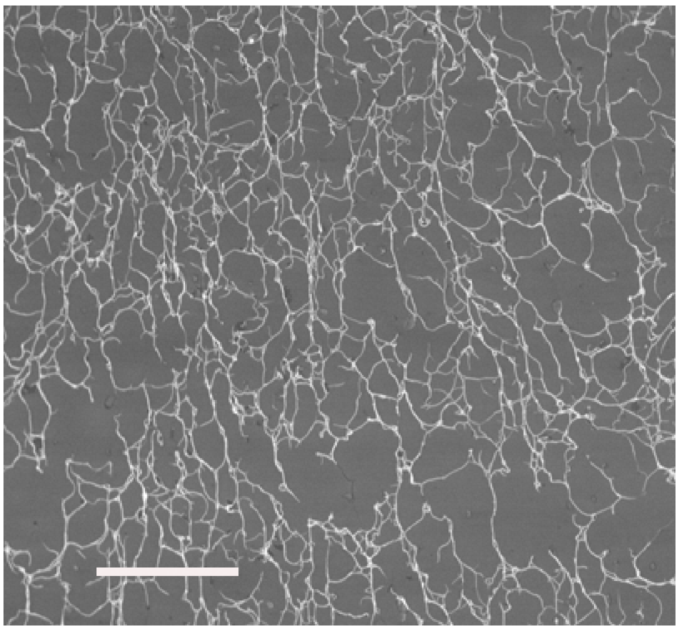

Supplementary Figure 2. Density of SWCNTs in D_2O used for UV-vis-NIR absorption spectrum measurements. Scanning electron microscope (SEM) image of single-wall carbon nanotubes (SWCNTs) by dropping one drop of D_2O solution containing SWCNTs on a Si substrate. Scale bar, 10 μm.

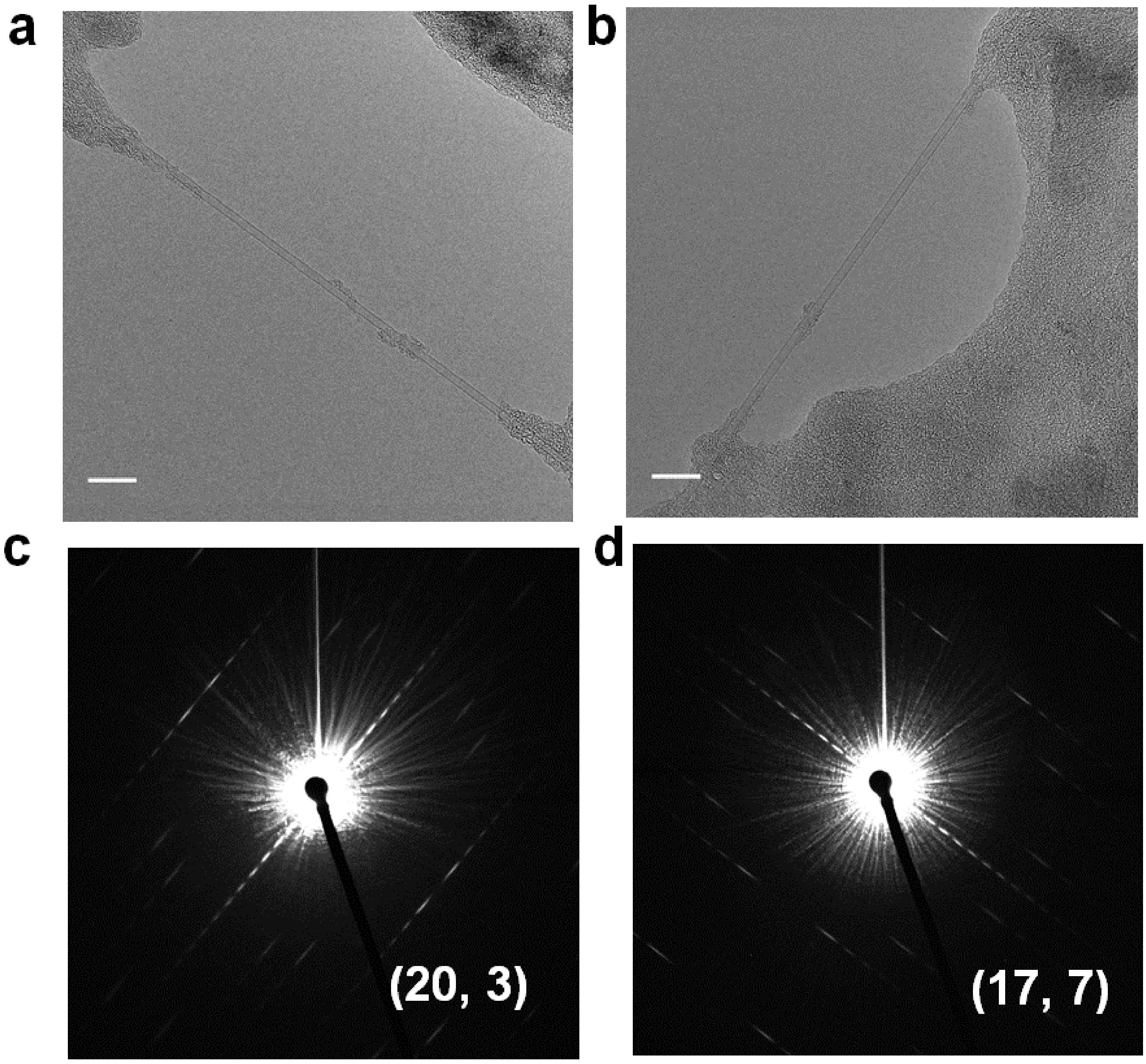

Supplementary Figure 3. Electron diffraction of SWCNTs. TEM images of two isolated SWCNTs (**a**-**b**) and their corresponding electron diffraction patterns (**c** and **d**) with chirality of (20, 3) and (17, 7), respectively. Scale bar, 10 nm.

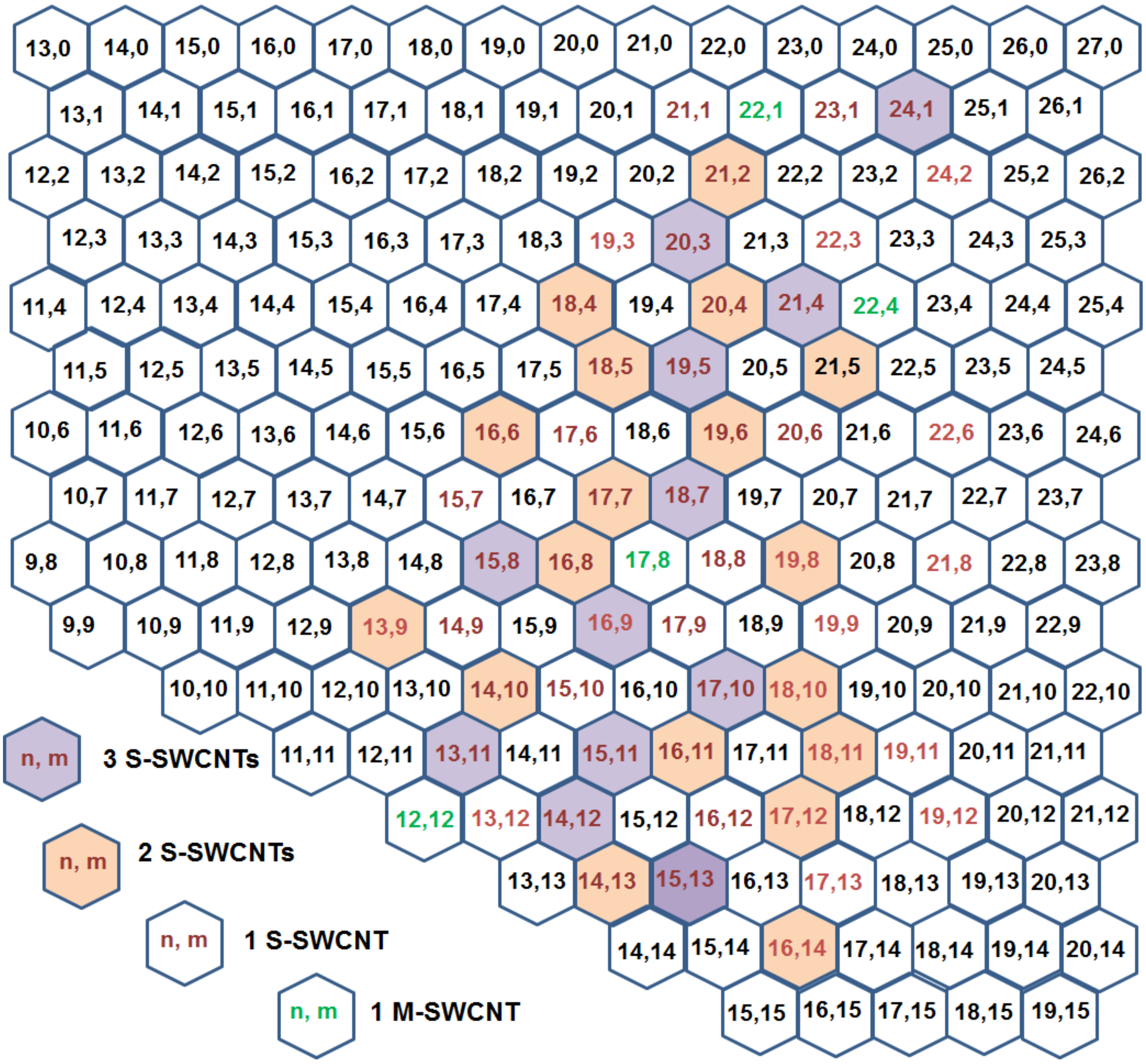

Supplementary Figure 4. Chirality indexes of the as-prepared SWCNTs. The chirality map of SWCNTs was determined from electron diffractions.

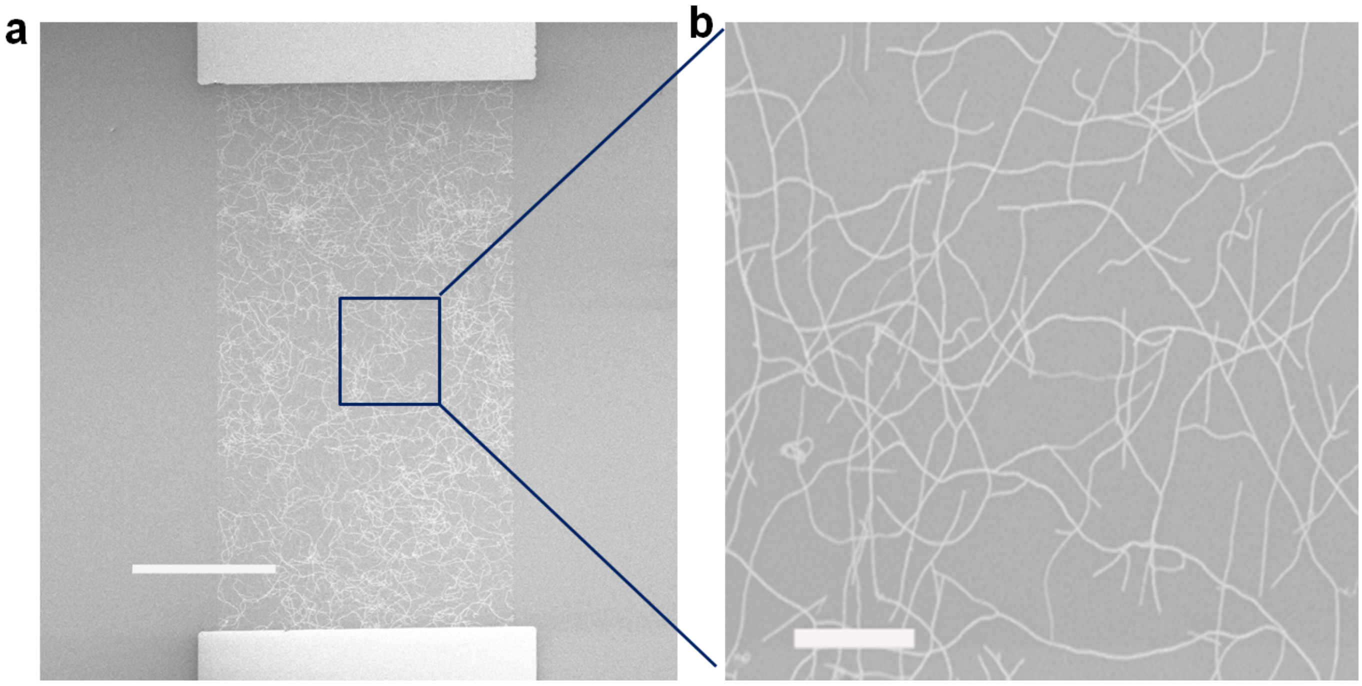

Supplementary Figure 5. Structure of a thin-film transistor (TFT) fabricated by using the SWCNTs as a channel material. (**a**) SEM images showing the configuration and morphology of the SWCNT-based TFT, in which the SWCNTs form a uniform network. Scale bar, 50 μm. (**b**) High magnification SEM image of the selected area in (**a**). Scale bar, 5 μm.

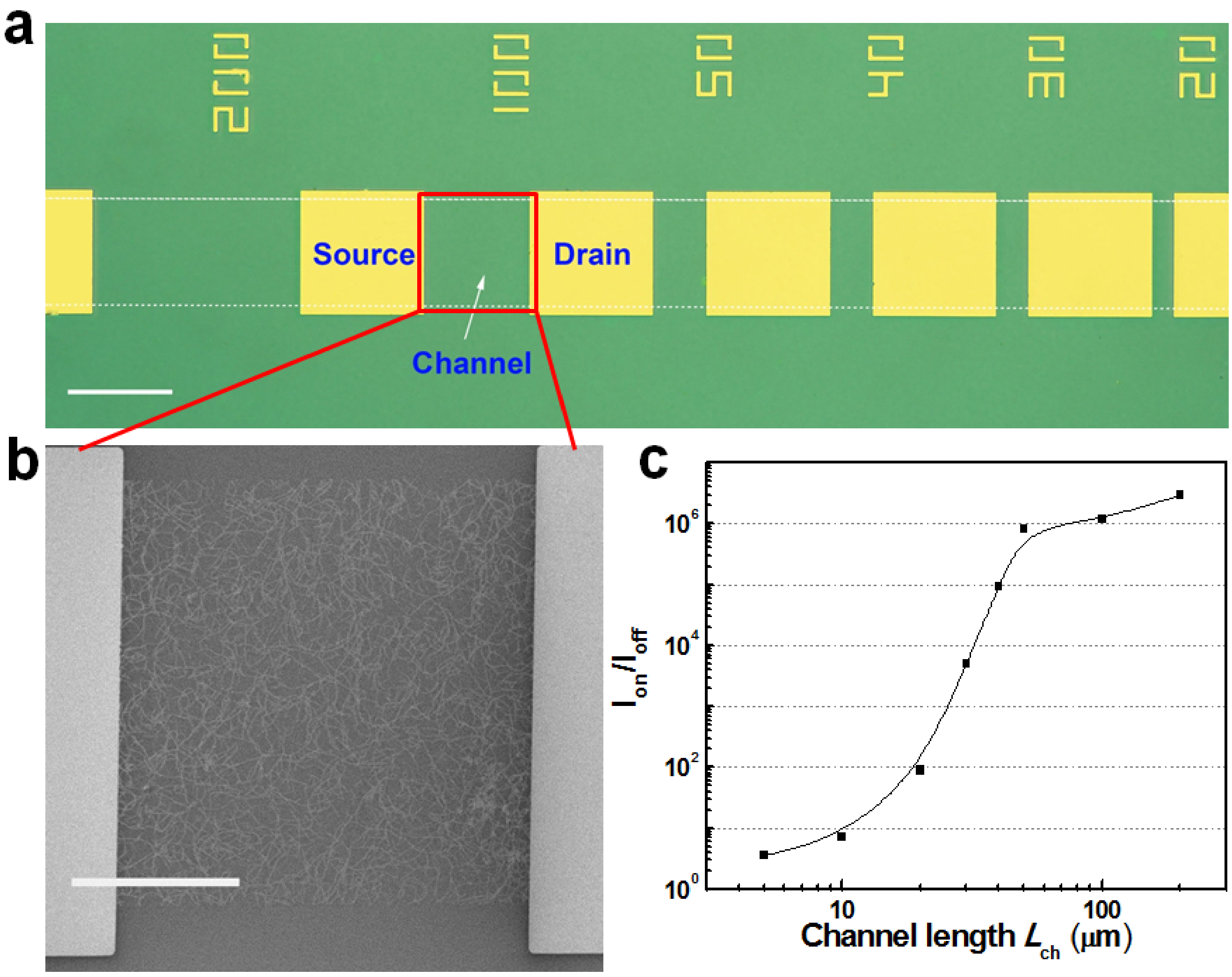

Supplementary Figure 6. Electrical performance of the fabricated SWCNT-based TFTs with different channel lengths. (**a**) Optical image of a series of bottom-gate TFTs with different channel lengths. (**b**) Typical SEM image of the channel of a SWCNT-based TFT with L_{ch} = W_{ch} = 100 μm. Scale bar, 40μm. (**c**) A plot of on/off ratio versus L_{ch}, and each solid square represents the median value measured from twenty-two TFTs.

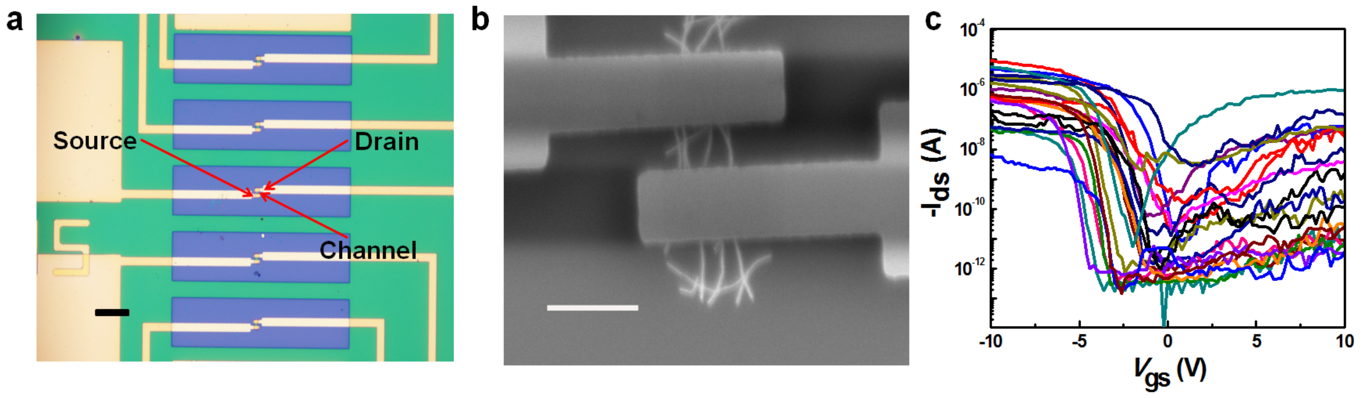

Supplementary Figure 7. Electrical performance of the short-channel TFTs. (**a**) Optical image showing the fabrication of SWCNT TFTs with a channel length and width of 1.5 and 2.5 μm, respectively. Scale bar, 10 μm. (**b**) SEM image showing a zooming view of a TFT device. Scale bar, 3 μm. (**c**) Transfer characteristics of 22 short-channel SWCNT TFTs.

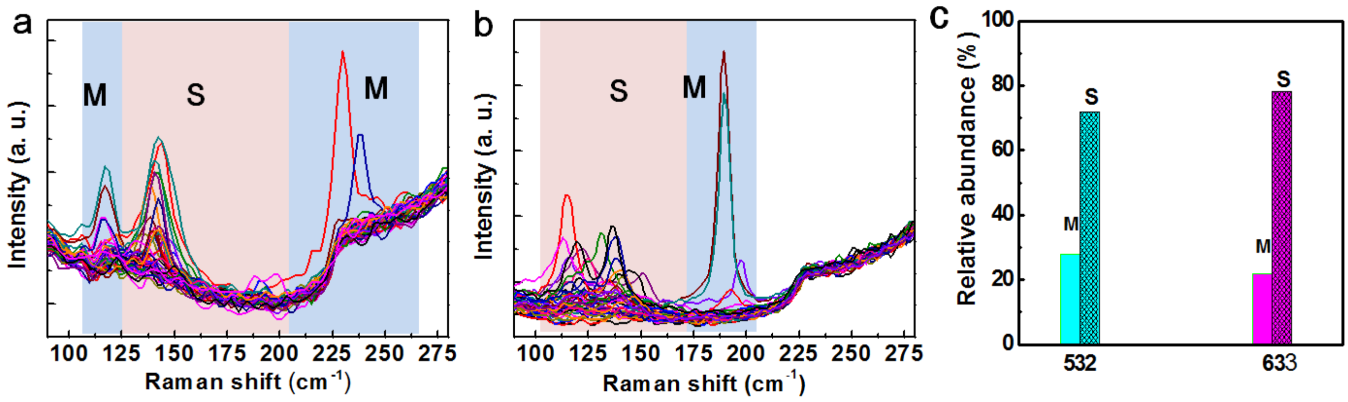

Supplementary Figure 8. Raman characterization of the SWCNTs grown from fully exposed Co nanoparticles. Radial breathing mode (RBM) peaks of the SWCNTs grown from fully exposed Co catalyst particles excited with (**a**) 532 nm and (**b**) 633 nm lasers. (**c**) The contents of m-SWCNTs and s-SWCNTs calculated from (**a**) and (**b**). The regions corresponding to semiconducting and metallic transitions are labeled as S (pink) and M (blue), respectively.

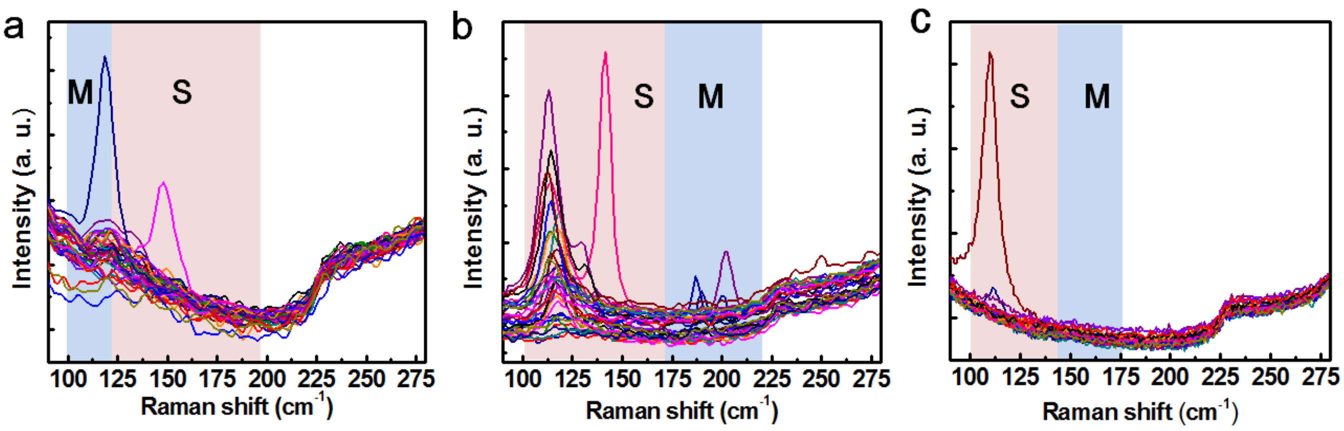

Supplementary Figure 9. Raman spectra of the SWCNTs with larger diameters. RBM Raman spectra of the SWCNTs grown from catalyst treated under a H_2 atmosphere at 800 °C for 10 min excited with (**a**) 532 nm, (**b**) 633 nm, and (**c**) 785 nm lasers. The regions corresponding to semiconducting and metallic transitions are labeled as S (pink) and M (blue), respectively.
